# Exploring the Demographic and Social Correlates of Burn Injuries: A Comprehensive Study From a Regional Burn Center in Pakistan

**DOI:** 10.7759/cureus.59619

**Published:** 2024-05-04

**Authors:** Nur Ul Ain, Dujanah S Bhatti, Muzna Mehmood, Husnain Khan

**Affiliations:** 1 Plastic and Reconstructive Surgery Department, Rawalpindi Medical University, Rawalpindi, PAK; 2 Plastic and Reconstructive Surgery Department, PostGraduate Medical Institute, Lahore, PAK; 3 Surgery Department, Rawalpindi Medical University, Rawalpindi, PAK

**Keywords:** electrical burn, thermal burn, burn wounds, burn grafting, burn center, injury epidemiology, acute burn, burn first aid, burn injury, burn

## Abstract

Burn injuries pose significant challenges to both patients and healthcare systems globally. This retrospective observational study, conducted at the burn center in a tertiary care hospital in Rawalpindi, Pakistan, aimed to delineate the patterns of burn injuries and correlate them with demographic and clinical presentations.

A total of 99 patients were included, with 54 males and 45 females, ranging in age from three months to 70 years. Flame burns were the most common type (n=69), with the majority being accidental (n=87). Limbs were the most frequently affected body parts (n=32), often with lesser-degree burns (n=28). Notably, self-inflicted injuries were predominantly observed in males (n=7), while assault cases were more common in females (n=4).

Statistical analysis revealed significant associations between the degree of burn and the body parts affected, as well as between the mode of injury and the affected body parts. Burn injuries due to assault or self-infliction tended to have higher morbidity rates, often resulting in fatalities. Additionally, the cause of burn injury showed significant associations with the affected body parts, with contact and electric burns affecting limbs and chemical burns mainly affecting the head and face.

These findings underscore the need for targeted burn prevention programs, emphasizing first aid education and addressing specific risk factors in high-risk groups and settings. By implementing preventive strategies and evaluating their effectiveness, the burden of burn injuries can be reduced, leading to improved patient outcomes and quality of life.

## Introduction

Burn injuries constitute a significant global public health challenge, characterized by tissue damage resulting from exposure to substances of elevated temperatures. These injuries often result in long-term ramifications, encompassing issues such as scarring, contractures, deformities, functional impairments, and psychological distress. The staggering toll is evident, with an estimated 180,000 deaths reported annually worldwide [1,2].

The management of burn victims is intricate, necessitating a profound comprehension of the injury's intricacies, encompassing its causative factors, severity, and associated risk elements. While the etiology, severity, and outcomes of burn injuries exhibit considerable diversity, certain determinants such as gender, age, mode of injury, and provision of initial aid profoundly influence the clinical trajectory and prognosis of affected individuals.

Existing research has illuminated various facets of burn injuries, including their patterns, risk factors, and outcomes. Studies have underscored the prevalence of flame burns and scald injuries, particularly among children, highlighting the differential impact of burn severity across various anatomical regions. Gender disparities in burn injury prevalence have been noted, with some studies indicating a higher incidence among males, while others report a higher prevalence among females. Additionally, the pivotal role of initial aid provision in mitigating burn severity and improving outcomes has been emphasized in numerous studies.

Socioeconomic factors are critical determinants of burn severity, often intertwined with household socioeconomic status. Studies consistently highlight a notable association between lower socioeconomic status and the increased severity and incidence of burn injuries [3].

For instance, research conducted by the World Health Organization (WHO) demonstrated a clear link between socioeconomic status and burn injury outcomes. Individuals from economically disadvantaged backgrounds were found to experience more severe burn injuries compared to those with higher socioeconomic status. This disparity is attributed to factors such as limited access to fire safety measures and unsafe living conditions [1].

Similarly, a meta-analysis examining data from various socioeconomic contexts supported these findings, revealing that lower socioeconomic status is associated with heightened burn severity. Factors such as overcrowded living conditions and inadequate access to healthcare contribute to the increased risk of severe burns among marginalized communities. Given the multifaceted nature of burn injuries and their complex etiology, comprehending the demographic and social correlates associated with these injuries is imperative for devising targeted prevention and intervention strategies. By elucidating the risk factors for severe burn injuries and identifying vulnerable populations, healthcare practitioners can tailor their interventions effectively, thereby enhancing prevention and management efforts [4].

Hence, the objective of this study is to delineate the patterns of burn injuries and correlate them with patient demographic and social factors to assess the risk factors associated with severe burn injuries. Building upon the foundation laid by existing literature, this study aims to offer insights into the epidemiology of burn injuries, thereby informing the development of tailored prevention programs and interventions.

## Materials and methods

This retrospective observational study, conducted between October 2018 and December 2022 at the Department of Burn and Plastic Surgery of Holy Family Hospital, Rawalpindi, Pakistan, aimed to comprehensively understand the demographics and clinical characteristics of burn victims presenting at the emergency department. Ethical approval for the study was obtained from the Ethics Review Committee of Rawalpindi Medical University, ensuring adherence to ethical standards throughout the research process.

To gather data, the authors meticulously designed a structured questionnaire informed by pertinent literature, ensuring the comprehensive coverage of relevant variables. Employing the consecutive sampling technique, the participants were selected, and each participant provided written informed consent, elucidating the study's purpose and their voluntary participation.

The inclusion criteria for the participants were stringent, focusing exclusively on individuals who were identified as burn victims and had presented to the emergency department during the study period. Conversely, individuals not meeting these criteria were excluded from the study to maintain its focus and integrity.

Data collection was organized into two main sections: the first is the demographic information and other factors. The participants were queried regarding essential demographic details such as age, gender, and means of transport to gain insights into potential socioeconomic influences on burn incidents. The second is the clinical characteristics. This section delved deeper into the clinical aspects of burn cases, encompassing inquiries about the cause of the burn, severity (degree), affected body parts, the provision of first aid, outcomes, and the nature of the incident (accidental, assault, or self-inflicted). Such detailed information aimed to provide a holistic understanding of the circumstances surrounding each burn case.

Descriptive statistics, including frequencies and percentages, were employed to summarize the collected data, facilitating a clear portrayal of the study cohort. Furthermore, to discern any associations between socio-demographic variables and clinical characteristics, statistical analyses such as Pearson's chi-square test and Fischer's exact test were applied, with a significance level set at a p-value of <0.05. These analyses were conducted utilizing the Statistical Package for Social Sciences (SPSS, version 26.0, IBM SPSS Statistics, Armonk, NY), ensuring robustness and reliability in the interpretation of findings.

## Results

Descriptive results

**Figure 1:**
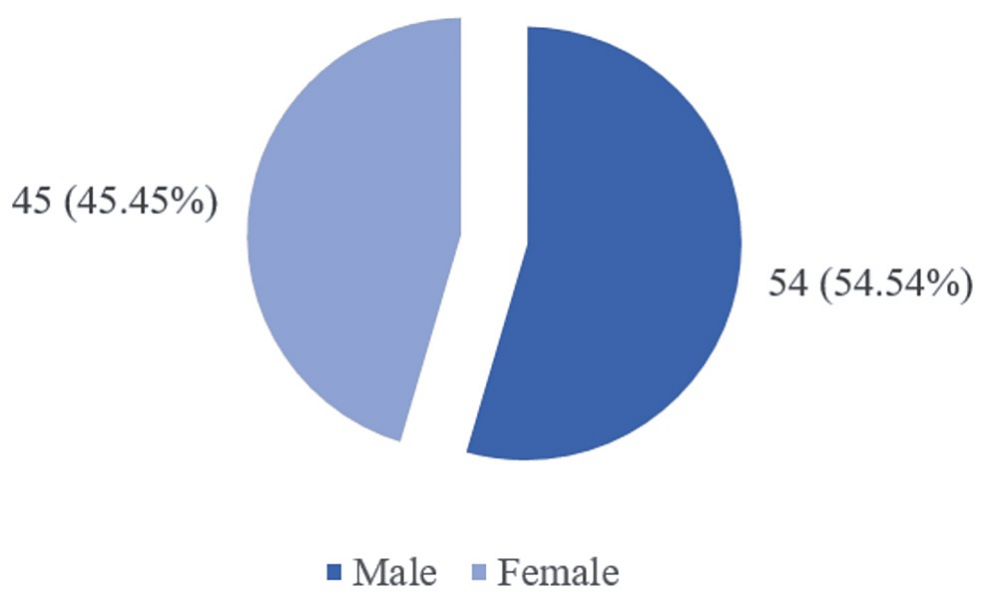
Gender distribution

Out of a total of 99 patients, our sample included 54 males and 45 females, having a mean age of 19.48±15.49 with an age range of three months to 70 years old (Figure 1).

Most of the patients suffered from flame burn injuries (n=69), and the injuries were accidental (n=87) for a huge majority of cases. Most of the injuries were observed on limbs (n=32), followed by ones on both the thorax and limbs (n=25) with a p-value of 0.00. Also, most of the limb injuries were lesser-degree burns with n=28. Interestingly, most of the cases (n=67, 67.7%) were discharged, while 25 (25.3%) resulted in fatal outcomes. We also had seven patients with self-inflicted injuries (7.1%), and all of these were male subjects, while most of the assault cases were females (n=4) than males (n=1) (p-value of 0.012) as can be seen in Table 1.

**Table 1:** Socio-demographics and clinical characteristics of patients (N=99) ED: emergency department

Description		Frequency	Percentages
Transport	Ambulance	51	51.5
	Non-ambulance	48	48.5
Cause	Scald	24	24.2
	Electric burn	3	3.0
	Fire	69	69.7
	Chemical	2	2.0
	Contact	1	1.0
Body part	Head and face	1	1.0
	Thorax	6	6.1
	Limb	32	32.3
	Head, face, and thorax	3	3.0
	Head, face, and limb	12	12.1
	Thorax and limbs	25	25.3
	All body parts	20	20.2
Degree	<20	51	51.5
	>20	48	48.5
ED disposition	Admitted	7	7.1
	Discharged	67	67.7
	Death	25	25.3
First aid	Received	59	59.6
	Not received	40	40.4
Burn type	Accidental burn	87	87.9
	Assault	5	5.1
	Self-inflicted	7	7.1

Analytical results

There was a statistically significant association between the body parts affected and the degree of burn (p-value of 0.00). Burn injury with a total body surface area (TBSA) of over 20% mostly involved all body parts or the thorax along with limbs. However, burn injuries with a TBSA of less than 20% predominantly affected limbs alone. Similarly, the association of the mode of injury with parts of the body affected was statistically significant (p-value of 0.00). Accidental burns chiefly involved limbs or the thorax and limbs simultaneously. On the other hand, self-inflicted and assault burn injuries primarily affected all body parts signifying the increased severity of such modes of injuries (Table 2).

**Table 2:** Association between body parts affected, mode of burn, and degree of burn

Body part	Mode of injury				Degree of burn		Total
	Accidental burns	Assault		Self-inflicted	<20%	>20%	
All body parts	12	2	6		1	19	20
Head, face, and thorax	3	0	0		1	2	3
Head and face	0	1	0		1	0	1
Head, face, and limb	11	1	0		7	5	12
Limb	32	0	0		28	4	32
Thorax	5	1	0		4	2	6
Thorax and limbs	24	0	1		9	16	25
Total	87	5		7	51	48	99

**Figure 2:**
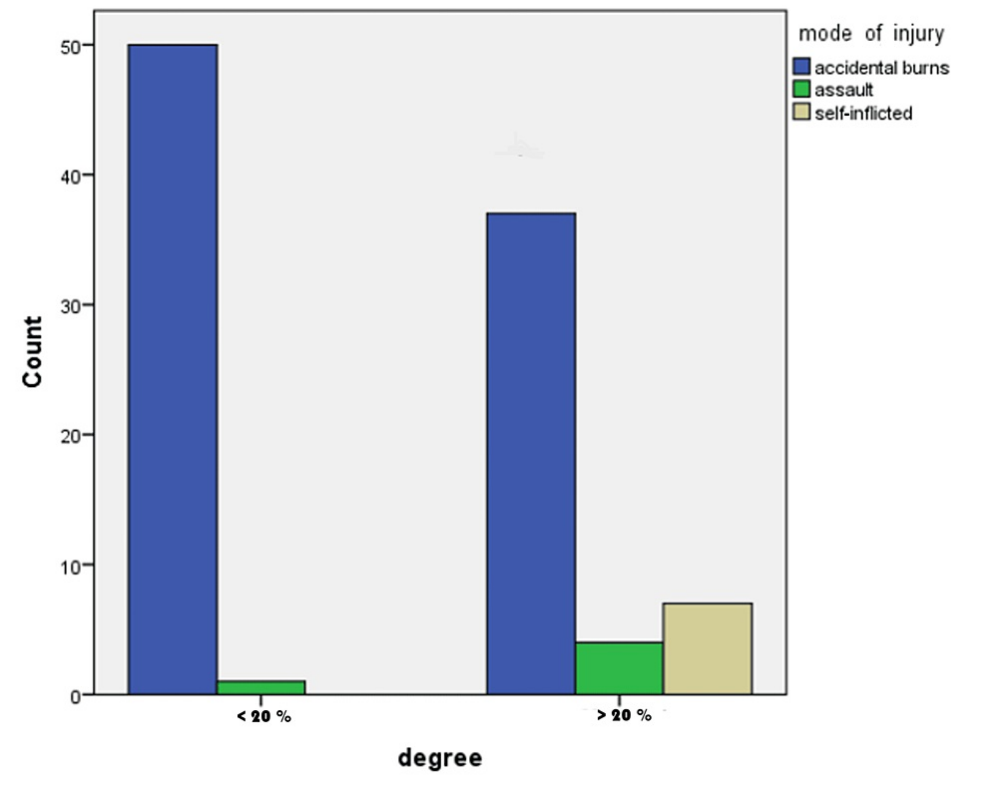
Relation of the mode of injury with the percentage of burn

Burn injuries due to assault tended to be mostly over 20% of TBSA, i.e., high-degree burn injuries (Figure 2). Also, all the self-inflicted burns were considered high-degree injuries (p-value of 0.01). However, six out of seven cases of self-inflicted injuries involved all body parts including the head and neck, face, thorax, limbs, and perineal region (p-value of 0.00).

For the association of the mode of injury with patient outcome in terms of emergency department disposal, the p-value was significant, i.e., 0.03. Most of the patients with burn injuries due to assault or self-infliction lead to death, which is a further evidence of increased morbidity associated with such injuries. However, the vast majority of accidental burns were discharged upon recovery. As depicted in the following table, four out of seven patients with self-inflicted wounds died; one was admitted, while two were discharged (p-value of 0.03). Three out of five cases of assault injuries resulted in death, while the other two were discharged (Table 3).

**Table 3:** Association of the mode of injury with patient outcome

Mode of injury	Outcome			Total
	Admitted	Death	Discharged	
Accidental burns	6	18	63	87
Assault	0	3	2	5
Self-inflicted	1	4	2	7
Total	7	25	67	99

For the association of the cause of burn injury with the body part affected, the p-value was significant, i.e., 0.02. Contact and electric burns mostly affected limbs; however, chemical burns mainly affected the head and face area in our study. Additionally, flame burns affected almost all the body parts (Table 4). We also observed that most of the scald burns (n=17) were also lesser-degree burns (p-value of 0.012).

**Table 4:** Association between the cause of burn injury and the body parts affected

Cause of burn injury	Body part								Total
	All body parts	Head, face, and thorax	Head and face	Head, face, and limb	Limb	Thorax		Thorax and limbs	
Chemical	0	0	1	1	0		0	0	2
Contact	0	0	0	0	1		0	0	1
Electric burn	0	0	0	0	3		0	0	3
Fire	19	3	0	9	19		3	16	69
Scald	1	0	0	2	9		3	9	24
Total	20	3	1	12	32	6		25	99

## Discussion

Burn is one of the leading causes of morbidity and mortality in the world [5,6]. Burn victims face difficulties not only physically but also economically, socially, and psychologically [7]. In this study, we present the burden and characteristics of burn injuries and their outcome in a burn center of a tertiary care hospital in Pakistan. Burns due to fire and scald burns from hot liquids were the most frequent causes of burn. Most of the burn patients received first aid prior to their arrival in the emergency department. Among various body parts, limbs were mostly involved. The majority of burn victims recovered and were discharged.

Similar to a number of studies, our study revealed male predominance in burn injuries [8-10]. This was in contrast to studies that reported burn injury more commonly in the female gender [11,12]. In our study, there was an obvious prevalence of self-inflicted burn injury in the male gender as also demonstrated by Pham et al. [13]. This was dissimilar to what was observed by Saaiq and Ashraf who found that the majority of victims of self-inflicted burns were females [14]. This contrast can be a result of various reasons such as the underreporting of self-inflicted injury in females due to delay in seeking care or inability to seek medical care due to social, psychological, and financial reasons. Furthermore, it was discovered that male burn victims with self-inflicted injuries were far more likely to die than male patients with accidental burn injuries, which corroborates the study done by Dobson et al. [15]. Most of the assault cases in our study were females as corroborated by a study on burn injury as interpersonal violence [16]. However, a study in South Africa reported that there was 1.5 times more risk for males to be the victims of burn assault than females [17].

Flame burn was the most common cause of burn injury, followed by scald burn, with the minority caused by exposure to chemicals or electricity. These findings were corroborated by several studies including those done by Tripathee and Basnet [18] and Karimi et al. [19]. Flame burn injury mostly occurs in adults, while children mostly fall victim to scald burns. However, in comparison, a study by Sharma et al. [20] reported electric burn as a more frequent cause of burn injury, which contrasts with a number of studies including ours [18,19].

The body parts most repeatedly involved were limbs either in isolation or with the involvement of other parts of the body, which is similar to the study by Siddiqui et al. [21], whereas the thorax alone or the head or face alone was involved less commonly. The predominating involvement of limbs can be attributed to their frequent involvement in daily activities and exposed position. There was a slight prevalence of those presenting with less than 20% TBSA of burn, which corroborated a retrospective analysis done in Southwest China [8].

Most of the patients presenting with burn injuries were discharged after complete recovery. Several studies reported similar findings [22,23]. It might be attributed to the fact that our study was conducted in a burn center designated for such cases and has healthcare workers experienced with treating burn victims. This was also observed in a study conducted by Mason et al. that depicted that the mortality rates after burn injuries were lower for patients treated at a burn center [24]. However, mortality rates after intentional burn injuries stayed remarkably higher [10]. This might be linked to the fact that intentional burn injuries have higher TBSA involved, associated inhalational injury, and increased depth of burn, all of which lead to a poor prognosis. Most of the patients have received first aid treatment, which played a role in their ultimate recovery as first aid helps in limiting burn injury.

In our study, we found that contact and electric burns primarily targeted the limbs, which was similar to the results of a retrospective analysis done by Gurbuz and Demir [25], while chemical burns predominantly affected the head and face region, which was in contrast with the findings of a study conducted in western Zhejiang Province, China, where upper extremities were predominantly affected [26].

The strength of this study is that it has been conducted in a high-volume burn center in a major city in Pakistan. Furthermore, a thorough examination of potential confounding factors was conducted. Moreover, the study adheres to the Strengthening the Reporting of Observational Studies in Epidemiology (STROBE) guidelines in its formulation and presentation. Also, there were some limitations in our study. Firstly, our study only included acute burn injuries presenting in the emergency department, and outpatient burn victims were excluded. Secondly, there is significant variation among physicians in the estimation of burn size, which might lead to the underestimation or overestimation of actual burn size. Moreover, the exact mechanism of the burn injury and the place where the burn occurred, socioeconomic status, and educational status were not mentioned in the patient files, and due to recall bias, they were not included. In view of our findings, we have deduced that it is crucial to educate individuals on basic first aid techniques, as their application can greatly improve outcomes. Additionally, tailored burn prevention initiatives should be developed for high-risk groups, with a focus on evaluating and implementing effective preventive measures. Children must be especially kept away from hot liquids as they are mostly susceptible to scald injuries due to negligence. For this, parents need to be educated about safer practices in daily routine, such as childproofing their homes, keeping hot liquids and matchboxes out of the reach of their child, and not drinking hot beverages with kids sitting in their laps as it can lead to accidental spillage. The use of protective clothing such as fireproof gloves and boots can protect most involved body parts, i.e., limbs, from burn injuries at the workplace.

## Conclusions

Our study demonstrates that the extent of the problem of burn injuries occurring in children at home and intentional burns in males requires greater efforts to prevent such injuries. People must be educated about first aid measures as using them could lead to a better outcome. Following this, burn prevention programs that address particular burn injury mechanisms in high-risk groups and high-risk settings need to be formulated, and preventive strategies need to be evaluated and employed.
